# CURRICULUM CONTENT FOR TRANSLATIONAL GEROSCIENCE EDUCATION: SOCIAL RESEARCH, POLICY, AND PRACTICE PERSPECTIVES

**DOI:** 10.1093/geroni/igae098.1339

**Published:** 2024-12-31

**Authors:** Richard Fortinsky

**Affiliations:** University of Connecticut, Farmington, Connecticut, United States

## Abstract

Geroscience is an exciting paradigm with the potential to positively disrupt the biological aging process in humans, thereby extending the healthy lifespan. This presentation will offer considerations from social research, policy, and practice perspectives that could be incorporated as topics into educational curricula for scientists planning to engage in geroscience-guided clinical trials. Regarding social research, methodological considerations include strengths and weaknesses of different research designs and how to choose outcome measures aligned with hypothesized mechanisms of action of geroscience-guided interventions. When considering how and where to initiate clinical trials, investigators will confront issues including how equity can be optimized when recruiting participants, how risks and benefits of participating in such clinical trials can be best communicated to potential participants, and how to balance scientific rigor with pragmatic concerns when determining inclusion and exclusion criteria. At a broader level, social research perspectives include how preferences for geroscience-guided therapies vary among individuals according to race, ethnicity, socioeconomic status, and degree of disability. Policy-related curriculum topics could include how geroscience therapies, if found to be effective, might affect health insurance programs such as original Medicare, Medicare Advantage, and Medicaid. For example, care management programs offered by Medicare Advantage programs might have to be modified to account for a beneficiary population with fewer medical comorbidities, and demand for Medicaid-funded nursing home and home and community-based services might be affected if multiple comorbidities could be prevented or alleviated. Practice considerations include how the healthcare workforce will be affected by the advent of geroscience-guided therapies.

